# Caspase1/11 signaling affects muscle regeneration and recovery following ischemia, and can be modulated by chloroquine

**DOI:** 10.1186/s10020-020-00190-2

**Published:** 2020-07-08

**Authors:** Ulka Sachdev, Ricardo Ferrari, Xiangdong Cui, Abish Pius, Amrita Sahu, Michael Reynolds, Hong Liao, Ping Sun, Sunita Shinde, Fabrisia Ambrosio, Sruti Shiva, Patricia Loughran, Melanie Scott

**Affiliations:** 1 Division of Vascular Surgery; Department of Surgery, University of Pittsburgh Medical Center, Magee Women’s Hospital, 200 Lothrop Street, Pittsburgh, PA 15213 USA; 2 McGowan Institute for Regenerative Medicine, University of Pittsburgh Medical Center, Bridgeside Point, Pittsburgh, PA 15213 USA; 3 Department of Pharmacology and Chemical Biology, University of Pittsburgh Medical Center, Biomedical Sciences Towe, Pittsburgh, PA 15213 USA; 4 Department of Hepatobiliary Surgery, Union Hospital, Tongji Medical College, Huazhong University of Science and Technology, Wuhan, 430022 China; 5 Department of Surgery 11/20/2018-11/19/202, Visiting scholar, University of Pittsburgh, Pittsburgh, USA; 6 Center for Biologic Imaging (CBI), University of Pittsburgh Medical Center, Biomedical Sciences Tower, Pittsburgh, PA 15213 USA

**Keywords:** Ischemic muscle, Inflammasome, Glycolysis, Oxidative phosphorylation, Lysosomal inhibition, Autophagy

## Abstract

**Background:**

We previously showed that the autophagy inhibitor chloroquine (CQ) increases inflammatory cleaved caspase-1 activity in myocytes, and that caspase-1/11 is protective in sterile liver injury. However, the role of caspase-1/11 in the recovery of muscle from ischemia caused by peripheral arterial disease is unknown. We hypothesized that caspase-1/11 mediates recovery in muscle via effects on autophagy and this is modulated by CQ.

**Methods:**

C57Bl/6 J (WT) and caspase-1/11 double-knockout (KO) mice underwent femoral artery ligation (a model of hind-limb ischemia) with or without CQ (50 mg/kg IP every 2nd day). CQ effects on autophagosome formation, microtubule associated protein 1A/1B-light chain 3 (LC3), and caspase-1 expression was measured using electron microscopy and immunofluorescence. Laser Doppler perfusion imaging documented perfusion every 7 days. After 21 days, in situ physiologic testing in *tibialis anterior* muscle assessed peak force contraction, and myocyte size and fibrosis was also measured. Muscle satellite cell (MuSC) oxygen consumption rate (OCR) and extracellular acidification rate was measured. Caspase-1 and glycolytic enzyme expression was detected by Western blot.

**Results:**

CQ increased autophagosomes, LC3 consolidation, total caspase-1 expression and cleaved caspase-1 in muscle. Perfusion, fibrosis, myofiber regeneration, muscle contraction, MuSC fusion, OCR, ECAR and glycolytic enzyme expression was variably affected by CQ depending on presence of caspase-1/11. CQ decreased perfusion recovery, fibrosis and myofiber size in WT but not caspase-1/11KO mice. CQ diminished peak force in whole muscle, and myocyte fusion in MuSC and these effects were exacerbated in caspase-1/11KO mice. CQ reductions in maximal respiration and ATP production were reduced in caspase-1/11KO mice. Caspase-1/11KO MuSC had significant increases in protein kinase isoforms and aldolase with decreased ECAR.

**Conclusion:**

Caspase-1/11 signaling affects the response to ischemia in muscle and effects are variably modulated by CQ. This may be critically important for disease treated with CQ and its derivatives, including novel viral diseases (e.g. COVID-19) that are expected to affect patients with comorbidities like cardiovascular disease.

## Background

Ischemia from peripheral arterial disease (PAD) leads to muscle fibrosis, fatty infiltration, poor functional outcomes and chronic pain (Doukas et al. 2013). We have shown that chloroquine (CQ) decreased fat replacement histologically during the recovery of muscle from ischemic injury in a mouse model of limb ischemia. It also increased caspase-1 cleavage/activation (Xu et al. 2018). Both CQ and its derivative hydroxychloroquine (HCQ) share an excellent safety profile, improve wound healing in autoimmune and sclerotic disease, are associated with favorable lipid profiles and have recently been found to attenuate cardiovascular events in rheumatoid arthritis (Sharma et al. 2016; Morris et al. 2011; Group* TCHS 1991). Additionally, both CQ and HCQ are also posited as potential therapeutic options caused by the novel coronavirus, SARS-CoV-2, to manage COVID-19 disease (Huang et al. 2020) (Singh et al. 2020). ,Those suffering from the worst complications of COVID-19 are also the same population (>65y) who are likely to have concomitant cardiovascular disease with ischemic complications like PAD. Therefore, a thorough understanding of the potential effects of CQ on ischemic muscle is paramount to avoid unintentional adverse effects.

In response to pathogen-associated or damage associated molecular patterns (PAMPs/DAMPS), cytosolic pattern recognition receptors like Nod-like receptor (NLR) with a pyrin domain-containing protein-3 (NLRP3) and absent in melanoma 2 (AIM2) initiate inflammasome formation, which cleaves and thus activates caspase-1 (Sun et al. 2017a). In macrophages this results in release of proinflammatory cytokines, IL1β and IL18, as well as inflammatory cell death, or pyroptosis (Sun et al. 2017b; Kim et al. 2019; Wang et al. 2018). Cytokine storm, including release of IL1β and inflammatory cell death, has been shown to be important in the severe acute respiratory syndrome (SARS) outbreak in 2002–2003 (Shi et al. 2019). However, in contrast to macrophages, inflammasome/caspase-1/11 activation in hepatocytes and some other epithelial cells, is a protective mechanism that promotes autophagy and prevents cell death in sterile, hypoxia-reoxia injury (Sun et al. 2017b). The role of inflammasome and caspase-1/11 is not well understood in muscle, although muscle and liver share remarkable regenerative capacity in response to injury. We therefore hypothesized that caspase-1/11 would also be protective in ischemic skeletal muscle. Our previous studies showing positive effects of CQ on muscle histology, and evidence that CQ increased caspase-1 activity in myocytes support the hypothesis. Here, we evaluated caspase-1/11 mediated effects of CQ not only on histology, but also muscle function, MuSC fusion characteristics, and cellular respiration. Understanding this pathway is particularly relevant in those disease states where caspase-1/11 is expected to be elevated, and where CQ is proposed as a therapeutic.

## Materials and methods

### Reagents

The reagents used for these experiments were as follows: antibodies to total and cleaved caspase-1 (#ab138483; Abcam, Cambridge MA), antibody to myosin 4 monoclonal antibody (MF20; MAB4470; R&D Systems, Minneapolis, MN) antibody to microtubule associated protein 1A/1B-light chain 3 (LC3; #L8918, Sigma,Sigma-Aldrich, St. Louis, MO) DAPI (#ab228549; Abcam), Cy-3-conjugated secondary antibodies (#ab6939;Abcam); antibody to myosin heavy chain fast (#ab51263) and slow (#ab234431). Primary antibodies were used at 1:250 or 1:1000 for immunohistochemistry (IHC) or western blot (WB), respectively; CQ, DMEM (#D5030, Sigma;), glucose (#G8270, Sigma), pyruvate (#S8636, Sigma), phenol red (#P5530, Sigma), sodium chloride (#S5886, Sigma), oligomycin (#75351, Sigma), carbonyl cyanide p-(trifluoro-methoxy) phenyl-hydrazone (FCCP), rotenone (#R8875, Sigma), and antimycin-A (#A8674, Sigma); glutaMAX (#35050–061; Gibco,Thermo-Fisher Scientific, Waltham, MA), dimethyl sulfoxide (DMSO; #BP231 Thermo-Fisher-Scientific).

### Animals

Male C57Bl/6 J (wild type; WT) were purchased from The Jackson Laboratories (Bar Harbor, ME) and used at 10–12 weeks of age. Caspase-1/11 knockout (KO) mice were a gift from Dr. Richard Flavell (Howard Hughes Medical Institute, Yale University) and were bred in our facility. Male KO mice aged 8–12 weeks and weighing 20–30 g were used in experiments. Purchased and in-house bred mice were co-housed for 2 weeks prior to experimentation to mitigate microbiome differences.

### Femoral artery ligation (FAL)

Unilateral FAL was performed on the right hindlimb, and exposure of the vessels without ligation was performed on the left hindlimb, as previously described (Sachdev et al. n.d.; Sachdev et al. 2013; Sachdev et al. 2014; Xu et al. 2015). To perform the ligation, the femoral artery and deep branch at the top of the limb was exposed. The femoral artery was ligated proximal and distal to the origin of the deep branch, which was also ligated with surgical ties. The C57Bl/6 J strain tolerates arterial ligation of this nature very well, and frank digital necrosis is rare (Shireman and Quinones 2005; McEnaney et al. 2016). Instead, ischemic injury is initiated in the *tibialis anterior* (TA) muscle, which results in angiogenesis and muscle regeneration over the course of 2–4 weeks (Xu et al. 2015). All surgical procedures were performed after intraperitoneal injection of ketamine/xylazine, followed by continuous inhalational anesthesia with vital sign monitoring. All procedures conformed to the Guide for the Care and Use of Laboratory Animals published by the United States National Institutes of Health (available at http://grants.nih.gov/grants/olaw/Guide-for-the-Care-and-Use-of-Laboratory-Animals.pdf) and the policies of the Institutional Animal Use and Care Committee of the University of Pittsburgh. Animals were treated with intraperitoneal (IP) injection of reagent grade CQ (50 mg/kg) or sterile phosphate-buffered saline (PBS) buffer on the day of surgery, and every second day subsequent to the procedure until sacrifice. CQ dosing was based on its adjunctive use in cancer models and in clinical trials (Maes et al. 2014; Liang et al. 2012). CQ was given using an IP route to better regulate the dose (Boone et al. 2015). CQ and its derivatives have a slow onset of action and a long-half-life, which was the rationale for every other day dosing (Patil et al. 2015). In some experiments, WT mice were pre-treated with CQ or buffer with the same dosing regimen for 21 days before the onset of ischemia. Mice were then sacrificed 24 h after FAL for evaluation of autophagic parameters by transmission electron microscopy (TEM) and LC3 staining.

Twenty-one days following FAL, *tibialis anterior* (TA) muscles were harvested at the time of euthanasia and snap frozen in liquid nitrogen-cooled isobutene (Shireman and Quinones 2005; Contreras-Shannon et al. 2007). Serial sequential cross-sections were generated at 8 μm thickness. Immunofluorescence was performed for caspase-1, identifying both cleaved and whole, uncleaved protein. Images were captured using an Olympus IX-81 inverted microscope and Olympus imaging system (Olympus of the Americas, Center Valley, PA). Sections incubated only with secondary antibody were used to determine background staining. Standard hematoxylin and eosin (H&E) staining was done to assess myofiber size and fat replacement, and Masson’s trichrome staining was performed to document fibrosis. Immunohistochemistry was done to determine myosin heavy chain slow and fast twitch fiber composition. Semi-quantitative total caspase-1 expression, LC3 as well as myofiber cross sectional area was determined using Image J (ImageJ, U. S. National Institutes of Health, Bethesda, Maryland, USA, http://imagej.nih.gov/ij/, 1997–2016). As LC3 distribution was critical, integrated density was used to evaluate LC3 expression, which allows quantification of staining per unit area. Staining of slow and fast twitch fibers was quantified and counted as positive for brown and negative for blue. For all imaging, data from a total of a minimum of 12 images per animal (4 images/section) were averaged and compared across experimental groups.

#### Transmission electron microscopy (TEM)

For in vivo electron microscopy experiments, euthanized mice were whole body perfused with cold PBS, followed by 2% paraformaldehyde in 0.1 M phosphate buffer (pH 7.4), and then 2.5% glutaraldehyde. Samples were sectioned into 2 cm cubes, followed by post-fixation for 6 h in 1% osmium tetraoxide. Samples were washed three times with PBS and underwent serial dehydration with graded alcohols. Samples were then embedded in epoxy resin. Each 2 cm tissue cube was sectioned into 70 nm thin sections with a microtome (Ultracut R; Leica), mounted on copper grids and post-stained with 2% uranyl acetate and 1% lead citrate. Sections were then dried and analyzed using a JEM 1011CX transmission electron microscope (Jeol, Peabody, MA). Images were acquired digitally from a randomly selected pool of 10–15 fields from nonischemic and ischemic limbs from WT and KO mice treated with either CQ or PBS. Autophagosomes were identified based on double membrane vesicles distinct from mitochondria and sarcoplasmic reticulum, and myofibers were identified as bundles containing distinct myofibrils. The number of autophagosomes/myofiber was calculated at 30,000x magnification from five images per animal.

### Harvest of muscle satellite cells

Muscle satellite cells (MuSC) were harvested from the TA muscle following 21 days of ischemia induced by unilateral FAL. Muscle segments were subjected to digestion with dispase and collagenase, and MuSC were separated using a modified pre-plate technique as described (Kozakowska et al. 2015). MuSC were isolated from the slow adhering fraction using CD56-mediated magnetic activated cell sorting (MACS) (Agley et al. 2013) .Cells were confirmed to be of muscle origin using MF-20 staining (Sondag et al. 2014).

### Western blot

For in vitro experiments, cells were washed with PBS at the endpoint of experiments, collected in lysis buffer (Cell Signaling Technology) with PMSF and protease inhibitors and centrifuged at 16,000 *g* for 10 min, and supernatant collected. For in vivo experiments, frozen tissue was homogenized in lysis buffer and centrifuged at 16,000 *g* for 10 min, and supernatant was collected. Protein concentrations of the supernatants were determined with the BCA (bicinchoninic acid) protein assay kit (Thermo Fisher Scientific). SDS loading buffer was then added to the samples. Denatured protein samples were analyzed by 10 or 15% SDS–polyacrylamide gel electrophoresis and then transferred onto a polyvinylidene difluoride membrane at 250 mA for 2 h. The membrane was blocked in 5% milk (Bio-Rad) in TBS for 1 h and then incubated overnight with primary antibody in 1% milk in TBS overnight. Membranes were washed three times in Tris-Buffered Saline-Tween (TBS-T) for 10 min, incubated with horseradish peroxidase-conjugated secondary antibody for 1 h, and then washed three times for 10 min in TBS-T before being developed for chemiluminescence (Bio-Rad). Digitally acquired images of the Western blot were analyzed using the gel analyzer macro on Image J analysis program. Relative density for each protein was divided by that for actin, and expressed as the ratio for each strain and treatment group.

### Live-cell metabolic assay

Analysis of metabolic function was performed using Seahorse Analyzer as described (Ryan et al. 2016). MuSC were plated in a 96-well Agilent Seahorse plate (Agilent 101,085–004) and allowed to adhere overnight. On the day of the experiment the media was replaced with 180 μL of DMEM containing 25 mM glucose, 1 mM pyruvate, 2 mM glutaMAX, 30 mM sodium chloride, and 40 μM phenol red, pH adjusted to 7.4. The cell plate was then incubated for 1 h at 37 °C for temperature equilibration. An Agilent sensor cartridge was then prepared with the following injections into ports A-D: DMEM as stated above, oligomycin A 2 μM, carbonyl cyanide p-(trifluoro-methoxy) phenyl-hydrazone (FCCP) 0.5 μM, and a mix of rotenone and antimycin A, both 2 μM. All reagents were solubilized in DMSO. After 1 h of incubation, the plate was loaded into a Seahorse XFe96 Analyzer (Agilent, Santa Clara, CA) to measure oxygen consumption rate (OCR) and extracellular acidification rate by XFe analyze. The instrument’s entire run time was 78 min, including calibration and equilibration of machine settings, 3 basal measurements followed by 2 measures for each injection port.

### Laser Doppler perfusion imaging

LDPI (PeriScan PIM III system, Perimed AB, Stockholm, Sweden) was performed under inhalational anesthesia to normalize hemodynamics at the time of each study. Placing the animals on a heating pad for the duration of the study maintained the body heat and animals were scanned in a prone position. Three scans were performed per animal, per time point, and the perfusion values for each limb were averaged. Results were expressed as the ratio of the ischemic to nonischemic limb perfusion for each time point (Limbourg et al. 2009).

### In situ functional muscle testing

Twenty one days following FAL, the force producing capacity of the ischemic TA muscle of control and experimental animals was evaluated using an in situ contractile testing apparatus (Model 809B, Aurora Scientific, Aurora, ON, Canada). Mice were anesthetized prior to surgery, and the peroneal nerve was exposed. The Achilles tendon was then severed in order to prevent any counteracting muscle response and the foot was secured to the footplate at a 20° angle. The electrodes were then placed on the exposed peroneal nerve after which the first single twitch stimulation protocol was conducted to measure peak twitch, time to peak twitch, and half-relaxation time. Next, the force-frequency curve was obtained by sequential administration of tetanic stimulations at 10, 30, 50, 80, 100, 120, 150, 180 and 200 Hz, with two-minute intervals between contractions. Results of both, single and tetanic stimulations were collected in torque (mN-m), and the absolute force values (mN) were evaluated by dividing torque by the length of the footplate (0.03 m). Specific force was then calculated by dividing the absolute force values by physiological cross-sectional area (CSA), calculated as the [muscle weight (mg)]/[muscle length (mm) × muscle density (1.06 mg/mm3)] (Egginton 1999).

### Statistical analysis

All data are expressed as mean plus/minus standard error of the mean (SEM). Analysis of variance (ANOVA) was used to compare multiple means in a pairwise fashion using the Holms-Sidak method available through SigmaStat (Systat Software Company, San Jose, CA). For comparisons between two groups, Student’s t-test was used to assess the means of continuous variables.

## Results

### CQ increases markers of autophagy in muscle

**Fig. 1:**
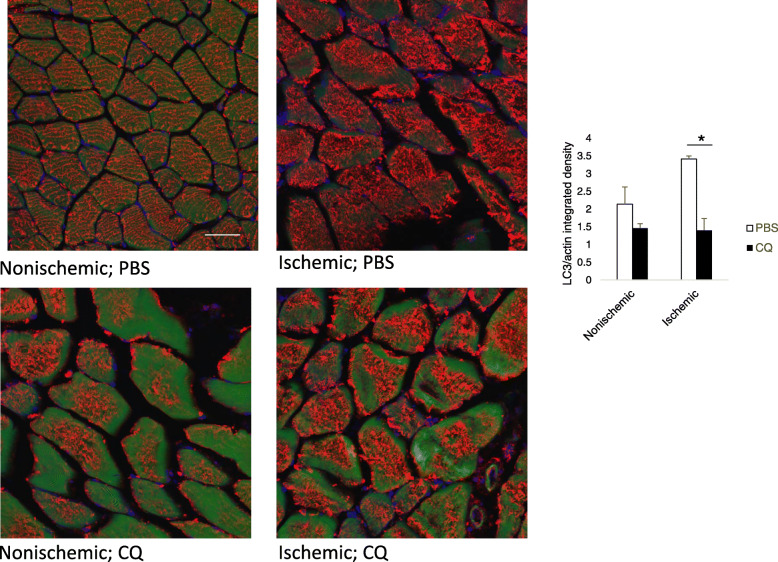
CQ consolidates LC3 expression in ischemic muscle. Immunohistochemical analysis of cross sections of nonischemic and ischemic *tibialis anterior* (TA) muscle from WT (C57Bl6/J) mice at 24 h after femoral artery ligation (FAL) treated with either PBS or CQ (50 mg/kg) for 21 days prior to unilateral FAL. LC3 expression (red); actin expression (green); DAPI nuclear stain (blue); pattern of LC3 expression (diffuse versus consolidated) analyzed using integrated density analysis (Image J) and expressed as a function of actin per unit area. Five images per limb/animal were evaluated. Representative images shown. *p < 0.005, PBS:CQ, ischemic limb *N* = 3 mice per treatment, 5 images/limb for each mouse; Scale bar = 10 μm

**Fig. 2:**
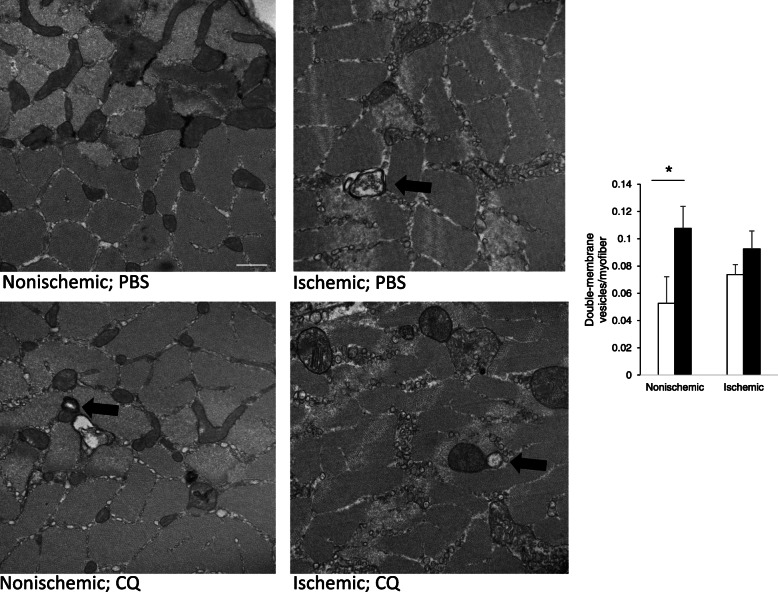
CQ increases double-membrane autophagosomes in nonischemic muscle. Transmission electron microscopy images of cross-sections of *tibialis anterior* muscle harvested from at 24 h from nonischemic and ischemic hindlimbs from WT C57Bl6/J mice treated with either PBS or CQ (50 mg/kg) for 21 days prior to the initiation of unilateral femoral artery ligation (FAL). Images captured at 30,000x magnification. Autophagosomes were identified as double membrane vesicles (arrows) and quantified as a function of the number of myofibers per image. A minimum of 5 images per limb/animal were evaluated. Representative images shown. *p < 0.05, PBS:CQ nonischemic limb, N = 3 mice per treatment, 5 images/limb for each mouse; Scale bar = 500 nm

CQ disrupts fusion of autophagosomes with lysosomes, thereby preventing the degradation of cytoplasmic proteins and organelles destined for degradation (Bae et al. 2008). We have previously shown that CQ halts autophagic flux in muscle allowing for accumulation of p62/sequestesome (SQSTM) which is a protein typically consumed by autophagy (Xu et al. 2018; Sachdev and Lotze 2017). During autophagosome formation, LC3 acquires a punctate, consolidated expression pattern, rather than a diffuse one, and since CQ does not prevent the formation of autophagosomes, but rather their degradation, CQ would be expected to result in auotphagosome accumulation, and therefore increased consolidated LC3 staining. We used integrated density measurements of LC3/actin staining to determine protein expression per unit area. Thus, a more diffuse pattern would be reflected in a higher integrated density, and reduced autophagosome formation. Here, we treated WT mice with CQ or PBS every other day before initiating FAL, and then sacrificed mice 24 h later to assess the effects of CQ and short-term ischemia on LC3 expression. CQ significantly reduced the integrated density of LC3/actin staining reflective of a consolidated pattern of staining, decreased autophagic flux and increased autophagosome accumulation in the ischemic limb (Fig. 1; *p* < 0.005, PBS:CQ). To confirm the accumulation of autophagosomes in more detail, we performed transmission electron microscopy to evaluate the presence of double-membraned vesicles consistent with autophagosomes. These were quantitated and expressed per myofiber. In nonischemic muscle harvested from the limb that did not undergo femoral artery ligation, CQ increased the expression of autophagosomes per cross sectional myofiber (Fig. 2; *p* < 0.05, PBS:CQ). In ischemic muscle, autophagosomes were elevated in both treatment groups, suggesting an increase in autophagic flux in response to ischemia.

### CQ increases caspase-1 expression in skeletal muscle

**Fig. 3:**
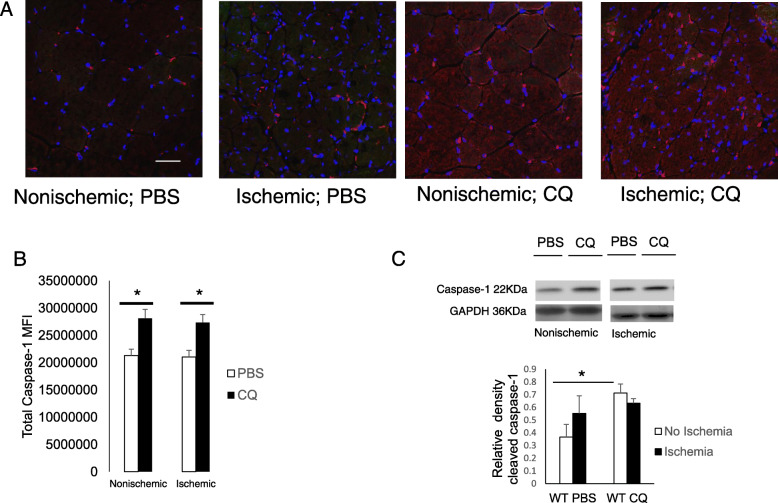
CQ increases caspase-1 expression in muscle. **a** Immunofluorescence of *tibialis anterior* (TA) muscle harvested 21 days after unilateral femoral artery ligation (FAL) in WT (C57Bl6/J) mice treated with PBS or CQ (50 mg/kg). Caspase-1 (red); DAPI nuclear stain (blue). Representative images shown (**b**) Caspase-1 mean fluorescence intensity (MFI) calculated after color separation using Image J analysis. * *P* < 0.01, PBS:CQ in WT and KO. **c** Western blots of cleaved caspase-1 and GAPDH loading control in whole muscle homogenates. Semi-quantitative analysis performed with Image J Gel Analysis of expression of cleaved caspase-1 relative to GAPDH. *p < 0.001 PBS:CQ, *N* = 4–5 mice per experimental group; Scale bar = 10 μm

We previously have shown that in isolated C2C12 myoblasts, CQ increases cleaved caspase-1 levels and caspase-1 enzymatic activity (Xu et al. 2018). One mechanism for this might be activation of inflammasome/caspase-1 with CQ-mediated accumulation of autophagosomes in the sarcoplasm due to disruption of autophagy. Having demonstrated that CQ similarly increases autophagosomes in muscle in vivo, we sought to determine what the effects of CQ were on caspase-1 expression after prolonged ischemia. Full-length and cleaved caspase-1 were detected immunohistochemically in TA myofibers from mice at 21 days after induced ischemia or normally perfused controls (Fig. 3a). Caspase-1 expression in muscle was increased significantly with CQ treatment with or without ischemia at 21 days. Interestingly, levels of caspase-1 measured using the same antibody were not altered in muscle by ischemia itself (Fig. 3a&b; *p* < 0.01 PBS:CQ nonischemic and ischemic limbs). Cleaved (activated) caspase-1 (22 kDa) trended an increase in TA of C57Bl/6 J mice after ischemia, and CQ treatment alone also increased this. Ischemia plus CQ treatment did not increase caspase-1 cleavage, however, suggesting that the effect of CQ is not synergistic with ischemia to increase caspase-1 activation (Fig. 3c; *p* < 0.001 PBS:CQ).

### Perfusion recovery at 21 days following femoral artery ligation is attenuated with CQ in WT but not in caspase-1/11KO mice

We have previously shown that following FAL return of perfusion in the limb rendered ischemic is about 60% after 21 days (Sachdev et al. 2013). when compared to the nonischemic limb. This is due to both an angiogenic and arteriogenic response, in which pre-existing yet underdeveloped collaterals respond by growth (Carmeliet 2000). Therefore we performed LDPI evaluation in both WT and caspase-1/11KO mice treated with/without CQ to determine whether the course of perfusion recovery was affected.

**Fig. 4:**
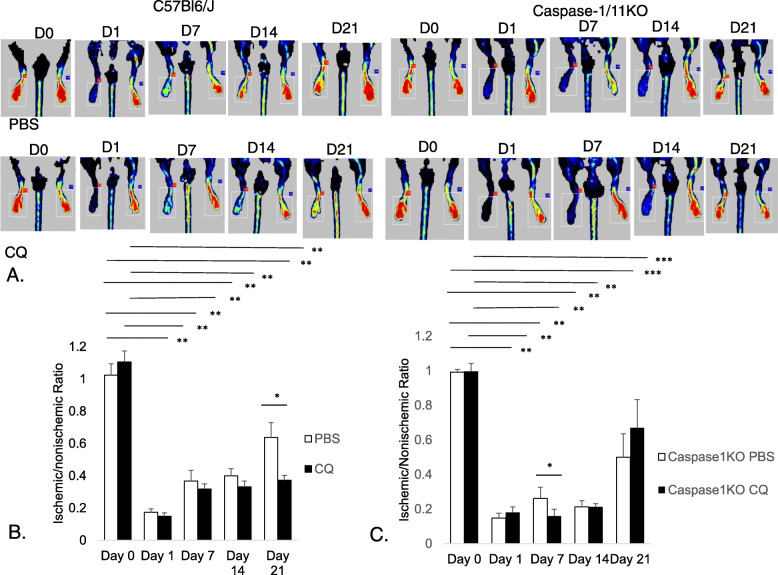
CQ reduces hindlimb perfusion recovery after femoral artery ligation (FAL). **a** Laser Doppler Perfusion Imaging (LDPI) of C57Bl6/J (WT) and caspase-1/11KO treated with either CQ or PBS (control) over the course of the study. LDPI was performed just before ligation (Day 0), and 1, 7, 14 and 21 days after FAL on the right hindlimb (screen left on each image). Representative images shown. LDPI in each animal expressed as a ratio of perfusion in ischemic to the nonischemic limb in. **b***N* = 7 WT mice/experimental group;*p < 0.01; **p < 0.001, and in (**c**) *p < 0.01; *p < 0.0001; ***p < 0.05 N = 4 KO mice/experimental group

At baseline, there was equal perfusion to both limbs between the WT and caspase-1/11KO mice with/without treatment with CQ (Fig. 4). Additionally, both strains demonstrated similar levels of ischemia the first day after ligation (Fig. 4a; *p* < 0.001 Day0:Day1). By 21 days, both WT and KO mice treated with PBS reached similar levels of perfusion recovery (Fig. 4b & c). In contrast, perfusion recovery in C57Bl/6 J mice treated with CQ was nearly half that of PBS treated mice (Fig. 4b; *p* < 0.01 CQ:PBS in WT). In caspase1/11KO mice, there was no significant effect of CQ on perfusion between PBS and CQ-treated mice after 21 days (Fig. 4c). These data suggest that caspase-1/11 does not affect arteriogenic/angiogenic responses after FAL, but that it may mediate detrimental effects on perfusion by CQ.

### CQ reduces fibrosis/fat replacement and regenerating myofiber size in WT but not caspase-1//1KO mice

Muscle regeneration after injury is a multi-step process that includes proliferation of MuSC, migration of those cells to injured areas, and fusion of cells to damaged myofibers to ultimately reconstitute muscle mass (Charge and Rudnicki 2004). During this process, fat deposition and fibrosis can also occur, which may affect functionality. Ischemic muscle in patients with peripheral arterial disease has been shown to be affected by this myopathy (Schieber et al. 2017), so understanding processes that can deter or improve regeneration in the setting of ischemia is important.

**Fig. 5:**
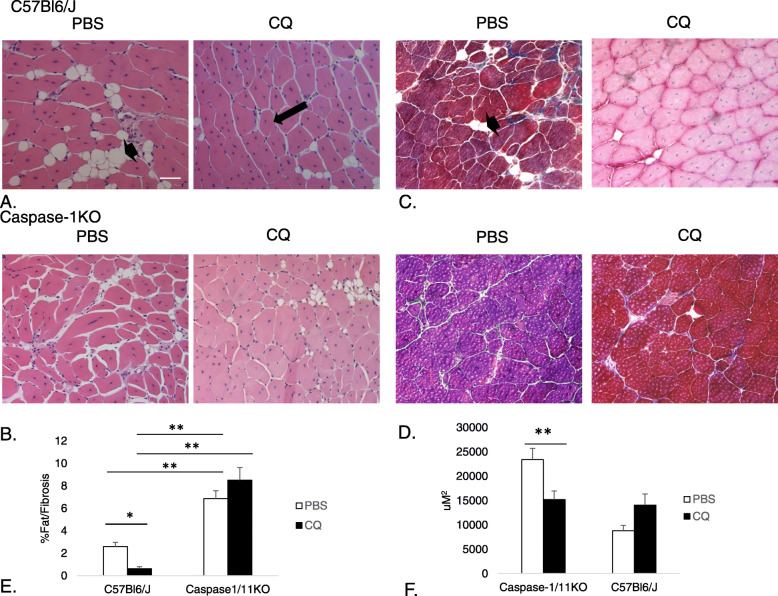
CQ reduces fibrosis and fat replacement in WT but not caspase-1/11KO mice. (**a** and **c**) Hematoxylin and eosin, and (**b** and **d**) Masson’s trichrome staining of *tibialis anterior* muscles from C57Bl6/J (WT) mice and caspase-1/11KO mice at 21 days after femoral artery ligation (FAL) and subsequent treatment with either PBS (control) or CQ (50 mg/kg) regenerating myofiber cross sectional area (CSA; arrow), fat replacement (arrowhead) and fibrosis (blue), respectively. **e** Quantification of CSA; *p = 0.003 PBS:CQ in WT; (**f**) Quantification of fat/fibrotic replacement of muscle tissue; **p < 0.001, PBS:CQ in WT. Representative images shown. N = 4–5 mice per experimental group

Similar to our previous study, mice rendered ischemic in one limb by FAL showed diminished fat and fibrotic infiltration in the ischemic limb with CQ administration ( (Xu et al. 2018); Fig. 5 a&c *p* = 0.003 PBS:CQ in WT). However, this presumed benefit was not seen in the absence of caspase-1/11 (Fig. 5b&d), and loss of caspase-1/11 signaling itself resulted in an increase in fat and fibrotic replacement within the tissue compared with WT, which trended worse with CQ treatment (Fig. 5e; *p* < 0.001 WT:caspase1/11KO). These data suggest caspase-1/11 may be protective against fat and fibrosis replacement of muscle after ischemia, and the protective effects of CQ may be dependent on caspase-1/11 in this context.

### Loss of caspase1/11 signaling results in abundance of fast-twitch but not slow-twitch myofibers in regenerating muscle

**Fig. 6:**
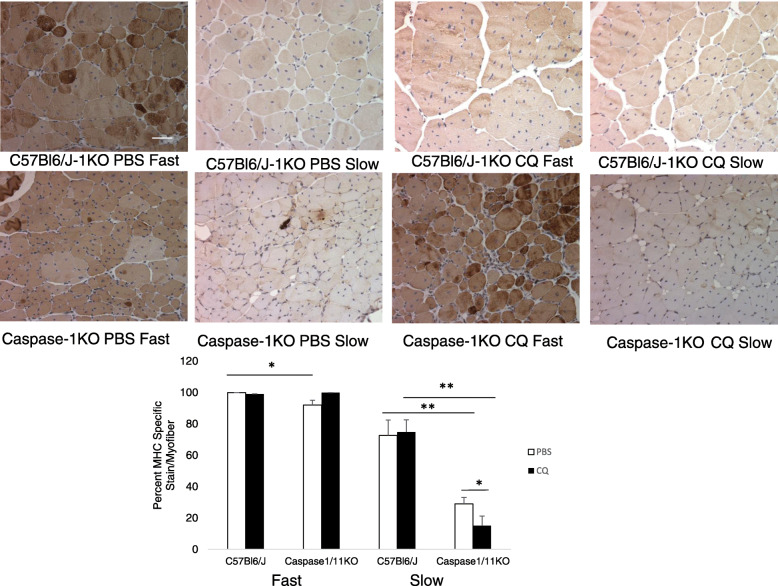
Loss of caspase1/11 favors regeneration of fast over slow twitch myofibers. Myosin heavy chain (MHC) specific for either fast or slow twitch myofibers in *tibialis anterior* (TA) muscle from C57Bl/6 J (WT) and caspase-1/11KO mice at 21 days after femoral artery ligation (FAL) treated subsequently with PBS (control) or CQ (50 mg/kg) (upper images); Quantification of fast and slow myofibers expressed as a fraction of total number of regenerating myocytes. Four images were analyzed per limb per animal. *p < 0.05, fast fibers in PBS in WT:KO, slow fibers in PBS:CQ in KO; **p < 0.001 slow fibers in PBS in WT:KO, slow fibers in CQ in WT:KO. N = 4 mice per strain per treatment group; Scale bar = 10 μm

Myofiber cross sectional area was also measured across the groups, which may be a reflection of either proliferative ability, hypertrophy or fusion of muscle satellite cells to existing fibers (Nielsen et al. 2017). Compared to PBS-treated WT mice, CQ treatment was associated with smaller regenerating myofiber size. Caspase-1/11KO mice also had smaller myofibers but the difference between PBS and CQ was not significant in knockout mice (Fig. 5f; *p* < 0.001 PBS:CQ). Differences in myofiber size might can indicate differences in muscle fiber type, where slow twitch fibers (Type I) are smaller, use primarily oxidative metabolism and are more likely to sustain long bouts of exercise due to greater efficiency, and where fast twitch fibers (Type II) are larger, use primarily glycolytic metabolism and are stronger and therefore able to sustain short bursts of exertion (Duan et al. 2017). In order to determine muscle fiber types, we performed specific staining to identify Type I or Type II myofibers. In contrast to our hypothesis, fast-twitch myofibers predominated in regenerating myocytes in WT mice regardless of whether the mice were treated with CQ or PBS. Fast twitch myofibers also predominated in caspase-1/11KO animals although to a slightly lesser degree than in control (Fig. 6; *p* < 0.05). However, unlike in WT mice, slow twitch oxidative fibers were nearly absent in caspase-1/11KO mice treated with PBS control. However, CQ increased the slow-twitch fibers in the knockout mice, suggesting a potential ability of CQ to promote fiber type switching in the absence of caspase-1/11(Fig. 6; *p* < 0.001 WT:KO; *p* < 0.05 PBS:CQ in KO).

### Caspase-1/11KO mice treated with CQ have lower peak contractions in response to tetanic stimulation

**Fig. 7:**
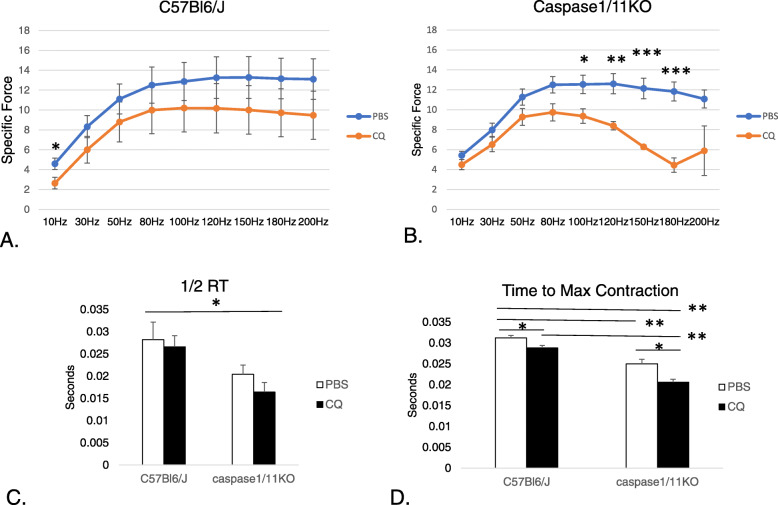
Loss of caspase-1/11 in addition to CQ results in diminished tetanic peak force Peak force measurements in peroneal nerve after tetanic stimulation at indicated frequencies (Hz) for each mouse in (**a**) C57Bl/6 J (WT) mice treated with PBS or CQ;*p < 0.05: PBS:CQ at 10 Hz, *N* = 5 PBS and 5 CQ B; or (**b**) Caspase-1/11KO mice treated with PBS or CQ; *p* < 0.05 PBS:CQ 100 Hz, **p < 0.01 PBS:CQ 120 Hz; ***p < 0.001 PBS:CQ 150 Hz and 180 Hz, N = 5 PBS and 5 CQ; (**c**) ½ relaxation time (RT) and (**d**) time to peak contraction in C57Bl/6/J and caspase-1/11KO after PBS or CQ treatment (C *p < 0.05; D *p = 0.003; **p < 0.001)

Our results so far have shown apparent histological differences between WT and KO mice and between effects of CQ treatments in regards to myofiber size, fat and fibrotic infiltration and fibertyping. To determine whether these had functional consequences, we performed in situ physiologic testing, which allows muscle contractile properties to be evaluated with an intact neurovascular bundle (Distefano et al. 2013). To perform the study, the peroneal nerve is stimulated using tetanic stimulation, and peak force as well as time to peak force and relaxation is measured. In situ testing revealed significant differences in peak tetanic force when comparing WT mice treated with PBS and those treated with CQ at baseline. Before the onset of tetanic stimulation at higher frequencies, CQ resulted in reduced peak force at 10 Hz (Fig. 7a; *p* < 0.05 PBS:CQ). In caspase-1/11KO mice, loss of peak force was most significant at higher frequencies of tetanic stimulation in those mice treated with CQ. Slow twitch fibers have lower peak force, and our findings were therefore consistent with the fibertype staining showing more slow twitch, weaker muscle fibers in caspase1/11KO mice treated with CQ but not PBS (Fig. 7b; *p* < 0.05 and 0.01; CQ:PBS in KO). Caspase1/11O mice treated with CQ had shorter half relaxation time (Fig. 7c; *p* < 0.05 CQ in KO: PBS in WT) and time to peak contraction as well (Fig. 7d; *p* < 0.05 PBS:CQ in WT and in KO, *p* < 0.01 PBS in WT: PBS in KO, CQ in WT:CQ in KO).

### CQ reduces MuSC myocte fusion index, which is exacerbated by the loss of caspase1/11KO mice

**Fig. 8:**
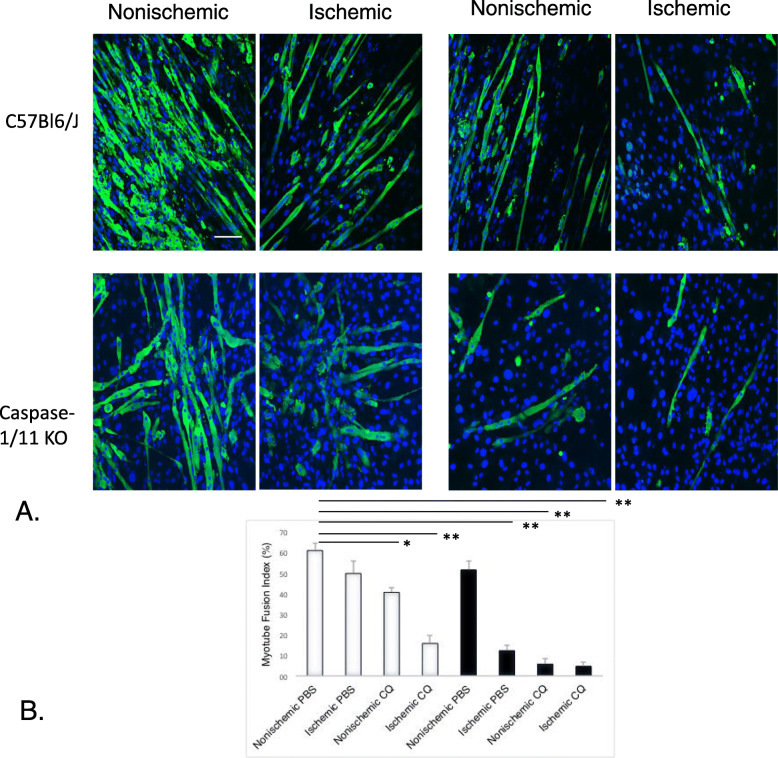
Myocyte fusion index (MFI) is attenuated in ischemia, CQ and absence of caspase-1/11 (**a**) Immunofluorescence of MF20 (green) and with DAPI (blue) in muscle stellate cells (MuSC) 5 days after harvest from *tibialis anterior* (TA) of nonischemic and ischemic limbs of C57Bl6/J (WT) and caspase-1/11KO mice at 21 days after FAL treated with either PBS or CQ. (**b**) Myocyte fusion index (MFI) quantified as number of nuclei within multinucleated fibers (> 3 nuclei) dividing by total number of nuclei. C57Bl6/J (WT) (white bars) and caspase-1/11KO (black bars) * *p* < 0.05, **p = 0.01, ***p < 0.001; N = 4 mice per strain per treatment group; Scale bar = 10 μm

Following injury, MuSC within muscle tissue proliferate, migrate to injured areas and fuse with damaged myofibers to promote regeneration (Charge and Rudnicki 2004). More fusion can result in larger myofibers. In order to evaluate the effects of CQ and caspase-1/11 on this process, MuSC harvested from the both ischemic and nonischemic muscle were cultured under conditions prompting their differentiation over the course of 5 days. Cells were then stained for MF20 (myocyte marker) and DAPI (nuclear stain) and the number of multinucleate fibers were quantified to provide a myocyte fusion index (MFI). MuSC from C57Bl/6 J and caspase-1/11KO both had 98% viability at harvest and baseline MFIs between WT and caspase-1/11KO cells were similar (Fig. 8a). MuSC harvested after ischemia had reduced MFI in WT compared with non-ischemic MuSC, but MFI was significantly reduced in MuSC from ischemic KO mice compared with non-ischemic control cells (Fig. 8a and b). CQ reduced MFI in WT mice, and this finding was exacerbated in caspase-1/11KO mice compared with PBS controls in MuSC from ischemic and non-ischemic mice (Fig. 8b; *p* < 0.001 to nonischemic, PBS WT). These data suggest lack of caspase-1/11 can synergize with CQ to reduce regenerative capacity in ischemic muscle.

### MuSC from caspase-1/11KO mice demonstrate an altered metabolic profile

**Fig. 9:**
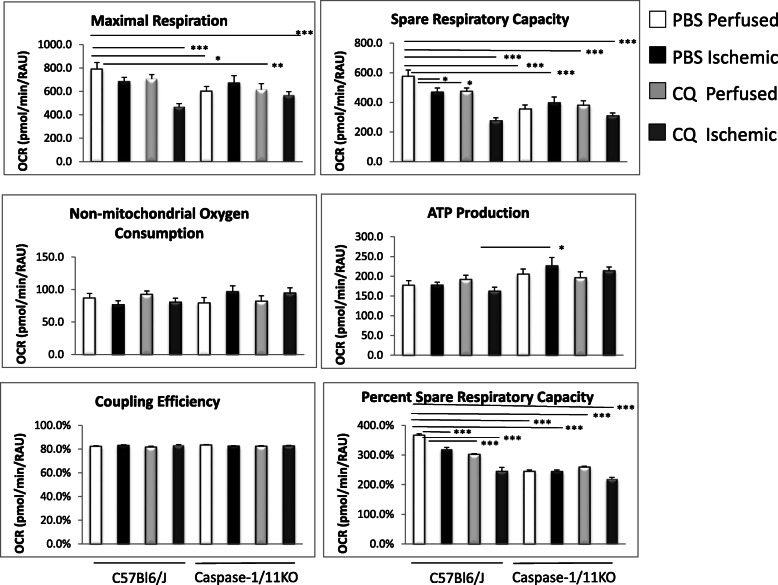
Ischemia, CQ absence of caspase-1/11signaling alter oxidative metabolism in muscle stellate cells (MuSC). Seahorse™ analysis of maximal respiration, spare respiratory capacity (including %), non-mitochondrial oxygen consumption, ATP production, and coupling efficiency in MuSC harvested from *tibialis anterior* (TA) of nonischemic and ischemic limbs of C57Bl6/J (WT) and caspase1/11KO mice at 21 days after femoral artery ligation (FAL) treated with either PBS or CQ (50 mg/kg); * *p* < 0.05, **p < 0.01, ***p < 0.001

Since our data showed differences in MFI, regenerating myocyte size and percent of oxidative muscle fibers between C57Bl/6 J and caspase-1/11KO mice, we sought to determine whether there were differences in metabolism governed by the presence of caspase-1/11 that may be further modulated by CQ. In order to evaluate this, we seeded MuSC on collagen-coated plates and evaluated both OCR and ECAR using a Seahorse™ Analyzer. Basal OCR and proton leak were similar among all the groups (not shown). Maximal respiration and spare respiratory capacity were highest in non-ischemic MuSC from perfused muscle in WT mice treated with PBS, and was reduced slightly in ischemia and to a significant extent in MuSC from non-ischemic mice treated with CQ alone, or CQ coupled with ischemia (Fig. 9). Absence of caspase-1/11 resulted in significantly diminished maximal and spare respiratory capacity compared to WT regardless of ischemia or CQ treatment. A similar pattern was seen for maximal respiration rate (Fig. 9). Interestingly, non-mitochondrial oxygen consumption and ATP production were similar between cells from WT and KO mice and across ischemic and CQ groups. These data suggest MuSC from caspase-1/11KO mice have diminished mitochondrial-dependent oxidative metabolism compared with WT cells, and this is not further diminished by CQ in contrast to WT.

### MuSC from caspase-1/11KO mice express high levels of enzymes involved in glycolysis

**Fig. 10:**
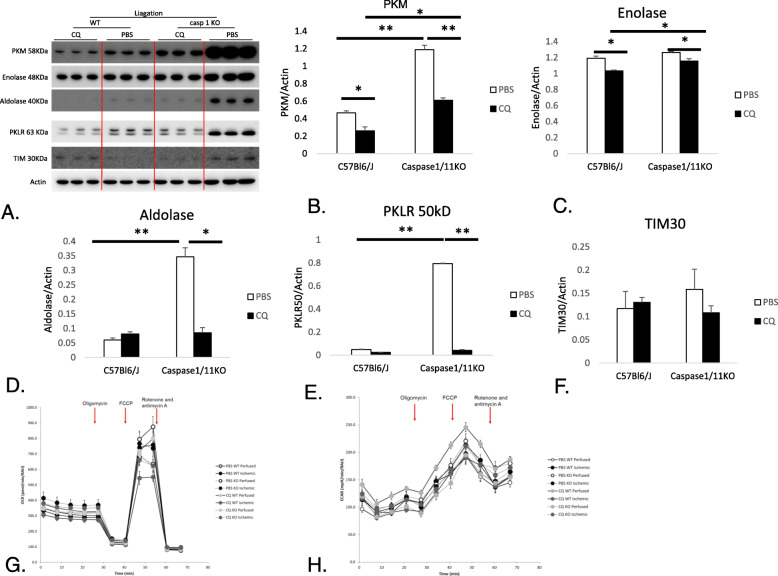
Glycolysis enzyme levels are elevated in caspase-1/11KO. **a** Western blot images of expression of Protein Kinase M (PKM), enolase, aldolase, Protein Kinase L/R (PKLR), TIM30 and actin loading control in muscle stellate cells (MuSC) at 5 days after harvest from tibialis anterior (TA) nonischemic and ischemic muscle at 21 days after femoral artery ligation (FAL) in C57Bl/6 J (WT) and caspase-1/11KO mice treated with PBS or CQ (50 mg/kg) (**b**-**f**) Quantification of relative expression of each enzyme relative to actin using Image J gel analyzer was used to quantify relative expression to that of actin loading control; (**b**) PKM *p < 0.03 PBS:CQ WT and CQ in WT:KO, **p < 0.001 PBS in WT:KO and PBS:CQ KO; (**c**) enolase *p < 0.03 PBS: CQ in WT and KO and CQ in WT:KO; (**d**) PKLR **p < 0.001 PBS:CQ in KO and PBS in WT:KO; (**e**) aldolase *p < 0.03 PBS:CQ in KO, **p < 0.001 PBS in WT:CQ; (**f**) TIM30; (**g**) oxygen consumption rate (OCR) and (**h**) extracellular acidification rate (ECAR) measured by Seahorse analyzer in MuSC from WT and caspase-1/11KO mice, *N* = 3 mice per strain per treatment

Others have reported that specific glycolytic enzymes are substrates for enzymatically active caspase-1, which may allow cells expressing higher levels of caspase-1 to shift their metabolic profiles towards mitochondrially-driven oxidative phosphorylation rather than more inefficient glycolysis (Shao et al. 2007). Our data above suggest that oxygen consumption was altered in caspase-1/11KO mice with reduced spare respiratory capacity and lower mitochondrial respiration caspase-1/11KO. CQ itself also markedly reduced spare respiratory capacity and mitochondrial respiration in WT mice, and effects were abrogated by the absence of caspase-1/11 signaling. We used Western blot of whole cell MuSC lysates from similar groups of mice to determine expression of key glycolytic enzymes (Fig. 10a), together with Seahorse™ analysis to measure extracellular acidification rate (ECAR), an indicator of glycolytic metabolism. MuSC from caspase-1/11KO mice treated with PBS had significantly elevated levels of PKM, aldolase and PKLR (Fig. 10b, d & e; *p* < 0.001 PBS WT:PBS KO) and expression of each of these glycolytic enzymes in caspase-1/11KO mice was significantly reduced by CQ (*p* < 0.03, PBS:CQ in KO). Levels of TIM30 and enolase were not different between WT and caspase-1/11KO-derived MuSC (Fig. 10c, f), but CQ also reduced the expression of enolase in both WT and KO mice (Fig. 10 c, *p* < 0.03 PBS:CQ in WT and KO). Graphs indicating OCR and ECAR are shown in Fig. 10g and h.

## Discussion

Our studies have relevance for patients who are suffering from muscle ischemia due to PAD, which affects a large number of patients and carries a significant threat of amputation in those who are not candidates for bypass or endovascular treatment (Marston et al. 2006). There are few effective medical treatments and so additional and alternative pharmacological attempts to improve perfusion and/or the response to ischemia may help attenuate the risk of limb loss in PAD patients. CQ and its derivative hydroxychloroquine (HCQ) have been suggested as potential treatments for PAD patients, and our previous studies showed similar potential protective effects in our mouse models. However, the exact mechanisms of action associated with many CQ and HCQ effects is not clear.

Recently, CQ and HCQ have been proposed as a potential protective treatment against the novel coronavirus, SARS-CoV-2, which causes COVID-19 disease (Shi et al. 2019). Since PAD affects a population primarily older than 65 years (Selvin and Erlinger 2004), this is also the population at high risk for infectious complications of SARS-CoV-2. PAD along with associated cardiovascular disease present significant risks for infected patients including contributing to the highest risk of death. Cytokine storm secondary to infectious activation of pathways including inflammasome and caspase-1/11 were shown to contribute to infectious complications of a similar coronavirus causing severe acute respiratory syndrome (SARS) in 2002–2003 (Shi et al. 2019). In this current study, as well as in our previous study, we show that CQ actually *increases* caspase-1 expression in muscle and this has both positive and detrimental effects. Our data suggest the need for caution using CQ and HCQ for COVID-19 patients without more fully understanding the pathways these drugs affect, particularly in end organ tissues such as liver and muscle.

Activation of both caspase-1 and caspase-11 can result in inflammatory cell death (pyroptosis) via cleavage of gasdermin D in monocytes and macrophages, which induces release of inflammatory cytokines (e.g. IL1β) and danger signals (Kayagaki et al. 2015) (Van Opdenbosch et al. 2014). However, in non-immune cells such as hepatocytes caspase-1/11 activation is protective in ischemic injury and promotes cell survival pathways such as autophagy (Sun et al. 2017b). Our data here suggest myocytes and hepatocytes share autophagy as an alternative function for caspase-1/11, with neither cell type being a major producer of caspase-1/11-mediated cytokines, IL1β and IL18, and both having excellent regenerative capacities.

Autophagy is likely to play multiple roles in recovery from muscle injury (Mammucari et al. 2008). Many studies suggest that autophagy is likely protective in skeletal muscle, preventing atrophy, and maintaining muscle mass (Masiero and Sandri n.d.). In inducible knockout mice lacking muscle-specific autophagy associated gene Atg7, muscle tissue was not protected from denervation-induced atrophy (Masiero and Sandri n.d.). CQ is known to inhibit autophagy by preventing autophagosome fusion with lysosomes, and it also effects lysosomal pH (Gallagher et al. 2017). Knowing the likely protective effect of autophagy on muscle, we were initially surprised by our previous finding that CQ resulted in less fat replacement in muscle tissue, suggesting protection rather than increased injury (Xu et al. 2018). Fat replacement and fibrosis may result from inflammation, and since CQ is known to be anti-inflammatory (Park et al. 2019) we hypothesized that CQ would reduce inflammatory markers, including those associated with inflammasome signaling, such as caspase-1. Instead, we found that CQ increased caspase-1 expression, cleavage and activity in C2C12 mouse myoblasts without increasing cell death (Xu et al. 2018). We therefore wondered whether caspase-1, which we knew can also induce autophagy and cellular protection (Cao et al. 2019) was influenced or influencing CQ effects in muscle related to autophagy, understanding that the effects would likely be both linked and complex. As caspase-1KO mice were not available to us, we used caspase1/11KO mice understanding the limitations of also knocking out caspase-11. We originally hypothesized that CQ-mediated increased caspase-1 in skeletal muscle would be protective, and that these protective effects of CQ would therefore be absent in caspase1/11KO mice. Our results demonstrated a more complex picture, with mixed effects of CQ on ischemic skeletal muscle, some of which were dependent on caspase-1/11 signaling, and some of which were independent.

In our previous report, we found that CQ disrupted autophagic flux resulting in accumulation of LC3IIB expression indicative of autophagosome formation, as well as decreased autophagic consumption of p62/SQTM (Xu et al. 2018). In our current study, CQ predictably resulted in an increase in autophagosomes in muscle, and also increased caspase-1 expression, including in between myofibers where capillaries and arterioles are found (Henning et al. 2019). In contrast to our hypothesis of protective effects of CQ in ischemic skeletal muscle, CQ reduced perfusion recovery after 21 days in WT mice but not caspase-1/11KO mice. Perfusion recovery in the murine model of hindlimb ischemia is dependent on arteriogenesis and angiogenesis (Carmeliet 2000; Buschmann and Schaper 2000) suggesting CQ effects on perfusion were caspase-1/11-dependent. Others have shown that inhibition of caspase-1 is associated with improved perfusion in a mouse hind-limb ischemia model, strengthening the idea that a CQ-induced increase in caspase-1 might ultimately be detrimental to perfusion (Lopez-Pastrana et al. 2015). We did not assess endothelial caspase-1 levels, and it is possible that these are important in the reperfusion effects of CQ. Further studies will be needed to elucidate the cell type responsible.

In other ways, however, our hypothesis of CQ protection was supported by our data. CQ reduced fat replacement and fibrosis in ischemic skeletal muscle in WT mice, with CQ effects dependent on caspase1/11 signaling in ischemic muscle at least in regards to histologic recovery. Additionally, CQ resulted in smaller myocytes in WT mice but not caspase1/11KO mice compared with PBS controls, which were potentially reflected in the fusion ability of MuSC (Ganassi et al. 2018), which was notably diminished in both CQ and caspase1/11KO mice. Decreased myofiber size may also reflect myofiber typing differences, and our data suggested a significant role for caspase-1/11 in determining fiber type after ischemic injury. Specifically, loss of caspase-1/11 signaling resulted in the near absence of slow twitch fibers, which was partially reversed by the addition of CQ. This is the first report describing skeletal muscle fiber-typing dependency on the presence of caspase1/11 signaling and is an intriguing finding that will lead to future studies.

Our fiber-typing suggested that caspase-1/11KO mice lacked slow twitch fibers except in the presence of CQ, and these data were supported peak force measurements after tetanic stimulation (von Roth et al. 2013). CQ resulted in significantly lower peak force during tetanic stimulation in caspase-1/11KO mice, but not in WT mice. Preservation of slow twitch fibers is optimal for sustained exercise like maintenance of posture, and walking (Askew et al. 2005) and these are the main fibers affected in muscular dystrophies (Lloyd et al. 2019; Ciciliot et al. 2013). More study is required to understand if there are therapeutic benefits of preserving one fibertype over the other in ischemic disease, but our data clearly show potential effects of autophagy on fibertype and downstream functionality.

Different myofiber types have different metabolic profiles, and as caspase-1 has been shown to degrade glycolytic enzymes (Shao et al. 2007), this may have played a role in the differences seen in our model. Our studies were performed on *tibialis anterior* which is predominantly fast-twitch and glycolytic. Indeed, most of the regenerating fibers in WT stained positively for fast-twitch specific myosin heavy chain. However, addition of CQ in the caspase-1/11KO mice was associated with increased in slow twitch fibers over the baseline in the KO mice, which may indicate protective effects of caspase-1/11 for preserving slow twitch fibers. Differences in fiber-typing between WT and KO mice, matched metabolic status of MuSC in our model. Both CQ and absence of caspase-1/11 reduced parameters associated with mitochondrial respiration, which suggests damaged or malfunctioning mitochondria unable to be removed by mitochondrial autophagy.

Our study has some important limitations. The murine hind-limb ischemia model is not a perfect model of PAD which is a chronic disease that takes many years to develop. There are some elements that may be similar physiologically, in that mice undergoing FAL develop a robust collateral network, and may thus experience intermittent episodes of ischemia/reperfusion similar to patients. We have optimized ways to harvest muscle satellite cells from patients with PAD and are evaluating the effects of CQ/caspase-1 signaling on those cells for a more relevant dataset. Additionally, we performed the FAL on relatively younger, and exclusively male mice which is not an adequate reflection of the population of people who suffer from PAD. This was done because we have noted gender-based variability in responses to FAL in mice in past experiments. These differences would be very interesting to pursue in the future, particularly because potential gender-specific effects of CQ in ischemic muscle is clinically relevant; patients with PAD are not only older and but also include both genders. These variables will be areas of future study in our laboratory.

## Conclusion

In conclusion, we demonstrate that CQ has specific effects on ischemic muscle in male mice, some of which are mediated by increased caspase-1/11 expression and signaling. CQ reduces fat and fibrosis in ischemic tissue, which depends on intact caspase-1/11 signaling through an unknown pathway. However, CQ also reduces perfusion recovery in ischemic muscle and these effects may be mediated by caspase-1/11. CQ and its derivatives are used in a multitude of diseases in patients with cardiovascular comorbidities, including PAD, therefore understanding the consequences of CQ treatment on a tissue specific level is paramount to improving patient outcomes to multiple disease processes.

## Data Availability

The datasets used and/or analyzed during the current study are available from the corresponding author on reasonable request.

## Abbreviations

AIM2: Absent in melanoma 2
CQ: Chloroquine
DAMP: Damage associated molecular patterns
ECAR: Extracellular acidification rate
FAL: Femoral artery ligation
HCQ: Hydroxychloroquine
KO: Knockout
LC3: Microtubule associated protein 1A/1B light chain 3
LDPI: Laser Doppler perfusion imaging
MuSC: Muscle satellite cell
NLRP3: Nod-like receptor (NLR) with a pyrin domain-containing protein-3
OCR: Oxygen consumption rate
PAD: Peripheral arterial disease
PAMP: Pathogenic associated molecular patterns
PKL/R: Protein kinase L and R
PMK: Protein kinase M
SARS: Severe acute respiratory syndrome
SCR: Spare respiratory capacity
TA: Tibialis anterior
TEM: Transmission electron microscopy
WT: Wild-type
